# Factors associated with infant mortality in Nepal: a comparative analysis of Nepal demographic and health surveys (NDHS) 2006 and 2011

**DOI:** 10.1186/s12889-016-3922-z

**Published:** 2017-01-10

**Authors:** Reeta Lamichhane, Yun Zhao, Susan Paudel, Emmanuel O. Adewuyi

**Affiliations:** 1 Department of Epidemiology and Biostatistics, School of Public Health, Curtin University, Perth, Australia; 2 School of Public Health, Faculty of Health Sciences, Curtin University, Perth, Australia; 3 School of Public Health, Curtin University, Perth, Australia; 4 Malteser International, Pulchowk, Laliltpur, Nepal

**Keywords:** Infant mortality, Region, Birth interval, Birth size, Breastfeeding, Nepal

## Abstract

**Background:**

Infant mortality is one of the priority public health issues in developing countries like Nepal. The infant mortality rate (IMR) was 48 and 46 per 1000 live births for the year 2006 and 2011, respectively, a slight reduction during the 5 years’ period. A comprehensive analysis that has identified and compared key factors associated with infant mortality is limited in Nepal, and, therefore, this study aims to fill the gap.

**Methods:**

Datasets from Nepal Demographic and Health Surveys (NDHS) 2006 and 2011 were used to identify and compare the major factors associated with infant mortality. Both surveys used multistage stratified cluster sampling techniques. A total of 8707 and 10,826 households were interviewed in 2006 and 2011, with more than 99% response rate in both studies. The survival information of singleton live-born infants born 5 years preceding the two surveys were extracted from the ‘childbirth’ dataset. Multiple logistic regression analysis using a hierarchical modelling approach with the backward elimination method was conducted. Complex Samples Analysis was used to adjust for unequal selection probability due to the multistage stratified cluster-sampling procedure used in both NDHS.

**Results:**

Based on NDHS 2006, ecological region, succeeding birth interval, breastfeeding status and type of delivery assistance were found to be significant predictors of infant mortality. Infants born in hilly region (AOR = 0.43, *p* = 0.013) and with professional assistance (AOR = 0.27, *p* = 0.039) had a lower risk of mortality. On the other hand, infants with succeeding birth interval less than 24 months (AOR = 6.66, *p* = 0.001) and those who were never breastfed (AOR = 1.62, *p* = 0.044) had a higher risk of mortality.

Based on NDHS 2011, birth interval (preceding and succeeding) and baby’s size at birth were identified to be significantly associated with infant mortality. Infants born with preceding birth interval (AOR = 1.94, *p* = 0.022) or succeeding birth interval (AOR = 3.22, *p* = 0.002) shorter than 24 months had higher odds of mortality while those born with a very large or larger than average size had significantly lowered odds (AOR = 0.17, *p* = 0.008) of mortality.

**Conclusion:**

IMR and associated risk factors differ between NDHS 2006 and 2011 except ‘succeeding birth interval’ which attained significant status in the both study periods. This study identified the ecological region, birth interval, delivery assistant type, baby’s birth size and breastfeeding status as significant predictors of infant mortality.

## Background

Infant mortality is defined as the death of a child before reaching the age of one in a specific year or period [1]. Early childhood is a vital period that determines their future health status. Therefore, infant mortality is a sensitive and important indicator that can be used to ascertain the physical quality of life index (PQLI) and wellbeing of a country [2, 3]. Infant mortality remains a major public health priority in many developing countries, and strategies aimed at addressing this challenge are of paramount importance. There are still many factors significantly associated with infant mortality that remain unexplored.

Globally, an estimated 4.6 million deaths occur annually during infancy, 99% of which occur in developing countries [1]. Global IMR has reduced to 34 deaths per 1000 live births in 2013 from an initial estimated 63 deaths per 1000 live births in 1990 [1, 4]. The infant mortality rate (IMR) has been declining steadily over the last century around the world; however, some developing countries like Nepal are still far behind. Despite the reduction in infant mortality by two thirds by many countries indicating a progress towards achieving the millennium development goal (MDG)-4 by the year 2015, this has not been evident in sub-Saharan Africa and some Asian countries including Nepal [5, 6]. Hence, disparities and inter-country variations still exist around the world in terms of IMR [7]. Recent trends of childhood deaths in African and Asian countries show that one out of every 12 infants does not survive until adulthood [8]. Additionally, global decline of child mortality is however dominated by the slow decline in sub-Saharan Africa [9].

In the last decade, Nepal made a substantial progress in many aspects of health care delivery; however, infant mortality remains a significant health challenge in the country [10–12]. Between 2006 and 2011 (a period of 5 years), only a marginal reduction was achieved in the rate of infant mortality in Nepal - from 48/1000 live births in 2006 to 46/1000 live births in 2011 [13, 14]. IMR in Nepal is higher in comparison to other Southeast Asian countries such as India, which has an IMR of 42 per 1000 live births; Bangladesh, 41 per 1000 live births and Sri-Lanka, 9 per 1000 live births [15–17]. Progress in IMR reduction is relatively slow when compared to other health indicators like maternal health and immunization of Nepal [10, 18]. Socioeconomic, demographic, ecological and other factors are associated with infant mortality in Nepal [10, 19]. In addition, there are inequalities in infant mortality within the country. For instance, most of the infant death occurs in Mountain region (73/1000 live births) [5], Far Western development regions (65/1000 live births) [13] and those residing in rural areas (47/1000 live births) [20].

Previous studies have explored the factors associated with infant mortality in Nepal. Khadka, Lieberman, Giedraitis, Bhatta and Pandey [20] reported the socioeconomic and proximate determinants associated with infant mortality in their recent study using NDHS. Similarly, Paudel Deepak, Thapa Anil, Shedain Purusotam Raj and Paudel Bhuwan [18] has analysed the trends and determinants related to neonatal mortality in Nepal using NDHS 2001 to 2011. Although many studies have been carried out previously to investigate factors contributing to infant mortality in Nepal using NDHS datasets of different surveys, to the best of our knowledge, there have been no studies conducted to compare the factors associated with the slow reduction in infant mortality between 2006 and 2011 using NDHS data. Hence, this study aims to explore the significant factors associated with infant mortality in 2006 and in 2011 in Nepal separately and then to fill the gaps by comparing the key factors associated with the slow reduction in infant mortality between 2006 and 2011 using two corresponding NDHS data.

## Methods

### Data sources

NDHS is a nationally representative survey conducted every 5 years in Nepal with the aim of providing reliable and up-to-date information on health and population issues in the country. It is a measure of the worldwide Demographic and Health Survey (DHS) project in the country. More precisely, DHS collects data on maternal and child health, reproductive health and fertility, immunisation and child survival, HIV and AIDS; maternal mortality, child mortality, malaria, and nutrition amongst women and children [21]. The datasets analysed in this study were extracted from the 2006 and 2011 NDHS. Furthermore, the study included only singleton live births born 5 years preceding both surveys. Both NDHS used multistage stratified cluster sampling technique. At first geographical areas were randomly selected, and then a complete list of dwellings and households were compiled. From those listed 20–30 households were selected using a systematic sampling procedure and then trained interviewers conducted household interviews with the eligible study population [13, 14].

In the 2011 survey, a total of 11,353 households were selected and 10,826 were successfully interviewed [13]. From these selected households, 12,674 eligible women (15–49 years) and 4323 eligible men (15–49 years) were successfully interviewed. Similarly, for the 2006 survey, a total of 8707 households were successfully interviewed out of 9036 selected households. Furthermore, 10,793 and 4397 eligible women and men of 15–49 years completed the interview, respectively [14]. The details of sampling instruments, sampling techniques, data collection and management used by NDHS have been previously discussed and published [4, 18, 20].

### Dependent variable

The dependant or outcome variable of this study is Infant Mortality. It has been defined as the probability of a child dying before the age of one (<12 months) in a specific year or period [1]. In regression analysis, the survival status of infants was further recoded as ‘1’ for infant who died within the first 12 months of life and ‘0’ for infants who survived beyond 12 months of life.

### Study framework and independent variables

**Fig. 1:**
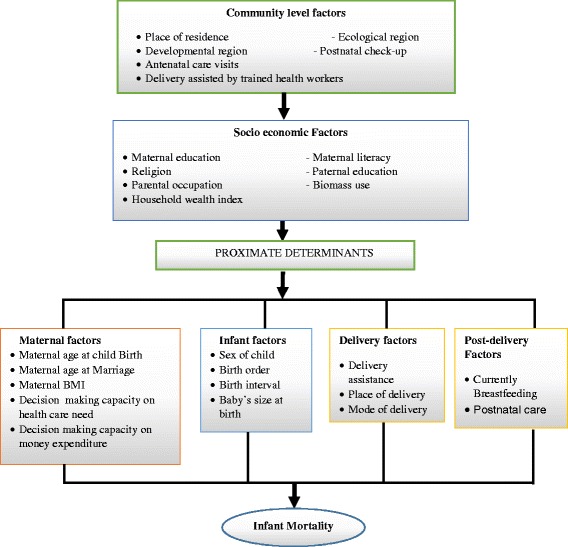
Conceptual framework for factors affecting Infant Mortality

In this study, modified version of Mosley and Chen’s [22] conceptual framework was used considering the context of Nepal (Fig. 1). Factors related to infant mortality were grouped into three levels, namely community factors, socio-economic factors and proximate factors. Mosley and Chen anticipated that proximate factors such as maternal, infant, delivery and post-delivery factors would directly influence infant mortality; and the socioeconomic and community level factors would have an indirect influence [22].

Selected independent variables along with definitions and coding categories are listed in Table 1.

**Table 1:** Operational definition and categorisation of selected explanatory variables for both NDHS 2006 and NDHS 2011

Variables	Definitions and category
Community level factors	
Development regions (Administrative)	Developmental regions (1 = Far-Western; 2 = Mid-Western; 3 = Western; 4 = Central; 5 = Eastern)
Ecological regions	Ecologically defined area. Division according to ecological zone (3 = Mountain; 2 = Hill; 1 = Terai)
Residence	Residence type (1 = Urban; 2 = Rural)
Antenatal care visits in the cluster	Any antenatal care service received by mother during pregnancy (0 = Yes; 1 = No)
Delivery assisted by trained health workers in the cluster	Birth assistance during delivery in the cluster (0 = Yes/Some; 1 = No/None)
Postnatal check-up/care received by mothers in the cluster	Postnatal check-up by mothers after delivery (0 = Yes; 1 = No)
Socio economic factors	
Maternal education	Maternal formal years of schooling/education (0 = No education; 1 = primary; 2 = secondary; 3 = Higher)
Paternal education	Father’s formal years of schooling/education (0 = No education; 1 = primary; 2 = secondary; 4 = Higher)
Religion	Religion of parents (1 = Hindu; 2 = Buddhist; 3 = Muslim; 4 = Christian/Kirat/other)
Maternal literacy	Mother’s literacy level (1 = able to read whole sentence or only parts; 2 = unable to read at all)
Paternal occupation	Father’s employment status (0 = Unemployed; 1 = Employed; 2 = Don’t know)
Maternal occupation	Mother’s employment status (0 = Unemployed; 1 = employed)
Wealth index	Household index of amenities/families economic status (1 = Poorest; 2 = Poorer; 3 = Middle; 4 = Richer; 5 = Richest)
Biomass use (cooking fuel)	Types of cooking fuel used in the family (1 = relatively non-polluting; 2 = relatively high polluting)
Proximate determinants	
Mother’s age at child birth	Maternal age at childbirth as categorical variable (1 = ≤16 years; 2 = 17–21 years; 3 = ≥22 years)
Mother’s age at marriage	Maternal age at first marriage as categorical variable (1 = ≤16 years; 2 = 17–21 years; 3 = ≥22 years)
Maternal Marital Status	Maternal marital status (0 = never married; 1 = Currently married; 2 = Widowed; 3 = Divorced/Separated)
Decision making on own health care need	Decision making capacity of mothers on her own health care needs (1 = Respondent alone; 2 = Respondent and husband/partner/other; 3 = Husband/partner alone; 4 = Someone else)
Decision making capacity on money expenditure	Decision making capacity of mothers on money expenditure. (1 = Respondent alone; 2 = Respondent and husband/partner/other; 3 = Husband/partner alone; 4 = Someone else)
Maternal BMI (kg/m^2^)	Maternal BMI as per WHO classification (1 = Underweight (<18.50); 2 = Normal range (18.50–24.99); 3 = Overweight/Obese- at risk (>25.0) ^a^Maternal BMI Asian (1 = Underweight (<18.49); 2 = Normal range (18.5–24.99); 3 = Overweight/Obese- at risk (>25.0)
Sex of child	Sex of infant (0 = Male; 1 = Female)
Birth order/rank	Birth rank of infant as a categorical variable (1 = 1^st^ birth rank; 2 = 2^nd^ or 3^rd^ birth rank; 3 = ≥4^th^ birth rank)
Birth interval	Succeeding birth interval (0 = ≤24 months; 1= > 24 months) Preceding birth interval (0 = ≤24 months; 1= > 24 months)
Baby’s size at birth (Birth size defined by baby’s birth weight)	Subjective assessment of the respondent on the baby’s birth size (1 = very large/larger than average (>3000 g); 2 = average (2500 to 3000 gm); 3 = very small/smaller than average (<2500 g))
Place of delivery	Delivery place (0 = Home; 1 = Health Facility)
Mode of delivery	Mode of delivery (1 = Non-caesarean section/Normal/vaginal; 2 = caesarean section)
Delivery assistant by	Type of delivery assistance (1 = Professionals (Doctors, Nurses and Midwives; 2-Traditional births attendants (TBAs); 3 = combined; 4 = No assistance)
Currently Breastfeeding	Breastfeeding status during the time of interview (1 = yes; 2 = No)

^a^Maternal BMI Asian was used in this study

### Statistical analysis

To adjust for unequal selection probability due to multistage cluster sampling, Complex Samples Analyses was used in the data analysis and modelling procedures. In the Complex samples analysis procedure, appropriate strata, cluster and weight variables were used to compute more accurate standard errors and confidence intervals. Three stage statistical analyses were conducted in this study. At the first stage, univariate analysis was carried out and IMR was reported, while at the second stage bivariate analysis assessed the unadjusted association (crude odds ratio (COR)) between infant mortality and each of the categorical predictors of interest using simple binary logistic regression analysis. All factors which were with a *p* value ≤ =0.1 (statistically significant at 10%) in the second stage were candidate factors for next stage multivariable regression analysis. In the third stage, a multiple logistic regression analysis was performed to assess the adjusted effect of factors on infant mortality for community level, socio economic level and proximate level factors, separately, and three sets of adjusted odds ratio (AOR) and its corresponding 95% confidence interval (CI) were reported.

Finally, an additional overall multiple logistic regression analysis was performed. The three sets of significant predictors obtained at the third stage modelling were entered into the final multiple regression model one after another from community level, then socioeconomic level and proximate level hierarchically. At each step of the modelling, the effect of these factors on the infant mortality was assessed and significant factors (at 5% level) were retained for next step of the modelling, using a stepwise backward elimination regression method. This hierarchical regression modelling process was repeated for 2006 and 2011 datasets separately.

The statistical analyses were carried out using IBM SPSS Statistics for Windows, Version 22.0 (IBM Corp. Released 2011. Armonk, NY: IBM Corp USA).

## Results

The study consisted of a total of 5836 live births with 280 infant deaths for NDHS 2006 and 5274 live births with 241 infant deaths for NDHS 2011. The unweighted IMR were 48 and 46 deaths per 1000 live births for NDHS 2006 and NDHS 2011, respectively.

### IMR for NDHS 2006 and 2011 (univariate analysis Tables 2 and 3)

**Table 2:** Infant Mortality Rate by selected background characteristics in the population of Nepal, 2006 (Total: 5836)

Community level Factors		
NDHS 2006		
Variables	IMR^a^	*P* value
Region		0.007
Far-Western	60	
Mid-Western	65	
Western	30	
Central	34	
Eastern	35	
Ecological Region (5836)		0.003
Terai	45	
Hill	30	
Mountain	78	
Residence(5836)		0.062
Urban	30	
Rural	43	
Maternal Antenatal care visits (2946)		0.001
Yes	17	
No	35	
Delivery assisted (4494)		0.013
Yes	40	
No	74	
Post-natal care (maternal) (734)		
Yes	21	
No	42	
Socioeconomic level Factors		
Maternal education (5836)		0.012
No education	48	
Primary	37	
Secondary	23	
Partner’s education (5836)		0.048
No education	53	
Primary	41	
Secondary	37	
Higher	25	
Religion (5836)		0.383
Hindu	43	
Buddhist	21	
Muslim	43	
Kirat	46	
Christian/others	51	
Maternal literacy (5836)		0.025
Able to read parts or whole sentence	33	
Unable to read at all	48	
Partner’s occupation (5836)		0.379
Unemployed	23	
Employed	42	
Don’t Know	22	
Maternal occupation (5836)		0.002
Unemployed	22	
Employed	48	
Biomass use (5836)		0.001
Relatively non-polluting	10	
Relatively high polluting	45	
Wealth Index (5836)		0.094
Poorest	52	
Poorer	38	
Middle	51	
Richer	30	
Richest	28	
Proximate level Factors		
Maternal Factors		
Mother’s age at Marriage (5836)		0.063
<16 years	46	
17-21 years	38	
>22 years	22	
Maternal age at Child Birth (5836)		0.104
<16 years	53	
17-21 years	42	
>22 years	30	
Mother marital status (5836)		0.532
Married	42	
Widowed	23	
Divorced/Separated	20	
Decision making on own health care need (5836)		0.007
Respondent alone	24	
Respondent & husband/partner/other	37	
Husband/Partner alone	53	
Someone else	46	
Decision making on own health care need (5836)		0.007
Respondent alone	31	
Respondent & husband/partner/other	56	
Husband/Partner alone	54	
Someone else	135	
Maternal BMI (Asian) (5836)		0.064
Underweight <18.49	46	
Normal range 18.5-24.99	42	
Overweight/obsess >=25.00	18	
Infant Factors		
Sex of Child (5836)		0.419
Male	39	
Female	44	
Birth order (5836)		0.004
1st birth rank	56	
2nd or 3rd birth	31	
>4th birth rank	43	
Preceding Birth Interval (5836)		0.001
<=24 months	57	
>24 months	26	
Succeeding Birth Interval (5836)		0.001
<=24 months	131	
>24 months	26	
Baby’s size at birth (4494)		0.013
Very large/Larger than average	34	
Average	37	
Very small/Smaller than average	64	
Delivery Factors		
Place of delivery (4496)		0.016
Home	45	
Health Facility	26	
Deliver Assisted by (4494)		0.001
Professional	22	
TBA	40	
Combined	113	
No assistance	74	
Mode of delivery (4494)		0.045
Normal (non-caesarean section)	42	
Caesarean Section	12	
Currently breastfeeding (5836)		0.001
Yes	34	
No	58	

Number and weighted numbers of infant and their respective percentages were calculated before calculating the infant mortality rate (IMR)

Unit: Death per 1000 live births

*Abbreviations*: *IMR* infant mortality rate

^a^Weighted

**Table 3:** Infant Mortality Rate by selected background characteristics in the population of Nepal, 2011 (Total: 5274)

Community level Factors				
NDHS 2011				
Variables		IMR^a^		*P* value
Region 5274)				0.582
Far-Western		54		
Mid-Western		42		
Western		47		
Central		43		
Eastern		37		
Ecological Region (5274)				0.001
Terai		41		
Hill		44		
Mountain		61		
Residence (5274)				0.022
Urban		40		
Rural		44		
Maternal Antenatal care visits (2994)				0.036
Yes		20		
No		38		
Delivery assisted (4183)			0.085	
Yes	42			
No	78			
Post-natal care (maternal) (2994)			0.189	
Yes	18			
No	26			
Socioeconomic level Factors				
Maternal education (5274)			0.356	
No education	49			
Primary	43			
Secondary	36			
Higher	28			
Partner’s education (5274)			0.010	
No education	57			
Primary	52			
Secondary	35			
Higher	21			
Religion (5274)			0.617	
Hindu	44			
Buddhist	58			
Muslim	30			
Kirat	0			
Christian/others	22			
Maternal literacy (5274)			0.017	
Able to read parts or whole sentence	36			
Unable to read at all	53			
Paternal occupation (5274)			0.905	
Unemployed	0			
Employed	44			
Don’t Know	40			
Maternal occupation (5274)			0.726	
Unemployed	46			
Employed	40			
Biomass use (5274)			0.050	
Relatively non-polluting	43			
Relatively high polluting	29			
Others	62			
Wealth Index (5274)			0.610	
Poorest	48			
Poorer	44			
Middle	45			
Richer	45			
Richest	30			
Proximate level Factors				
Maternal Factors				
Mother’s age at Marriage (5274)			0.092	
<16 years	49			
17-21 years	47			
>22 years	30			
Maternal age at Child Birth (5274)			0.466	
<16 years	48			
17-21 years	39			
>22 years	39			
Mother marital status (5274)			0.871	
Married	44			
Widowed	47			
Divorced/Separated	25			
Decision making on own health care need (5274)			0.370	
Respondent alone	45			
Respondent & husband/partner/other	37			
Husband/Partner alone	45			
Someone else	55			
Decision making on own health care need (5274)			0.455	
Respondent alone	25			
Respondent & husband/partner/other	29			
Husband/Partner alone	59			
Someone else	57			
Maternal BMI (Asian) (2557)			0.493	
Underweight <18.49				
Normal range 18.5-24.99				
Overweight/obsess >=25.00				
Infant Factors				
Sex of Child (5836)			0.212	
Male	47			
Female	40			
Birth order (5836)			0.272	
1st birth rank	51			
2nd or 3rd birth	40			
>4th birth rank	39			
Preceding Birth Interval (5836)			0.009	
<=24 months				
>24 months				
Succeeding Birth Interval (5836)			0.001	
<=24 months	151			
>24 months	41			
Baby’s size at birth (4494)			0.004	
Very large/Larger than average	29			
Average	41			
Very small/Smaller than average	70			
Delivery Factors				
Place of delivery (4496)			0.316	
Home	46			
Health Facility	38			
Deliver Assisted by (4494)			0.437	
Professional	39			
TBA	45			
Combined	36			
No assistance	78			
Mode of delivery (4494)			0.018	
Normal (non-caesarean section)	45			
Caesarean Section	11			
Currently breastfeeding (5836)			0.002	
Yes	36			
No	57			

Number and weighted numbers of infant and their respective percentages were calculated before calculating the infant mortality rate (IMR)

Unit: Death per 1000 live births

*Abbreviations*: *IMR* infant mortality rate

^a^Weighted

Tables 2 and 3 summarizes the IMR for the NDHS 2006 and 2011, respectively. The highest IMR was recorded in the Mountain region with 78 deaths per 1000 live births in 2006 and 61 deaths per 1000 live births in 2011. For both surveys, infants whose mothers had no education (74/1000 live births in NDHS 2006, 49/1000 live births in 2011), gave birth to her first child at the age younger than 16 years (53/1000 live births in 2006, 49/1000 live births in 2011) and did not have decision making authority on their own health care and money expenditure were found to have the highest IMR (Tables 2 and 3). In NDHS 2011, furthermore, IMR was also higher among infants who were born with preceding and succeeding birth interval of more than 24 months.

### Factors associated with infant mortality (third stage multiple logistic regression analysis Tables 4 and 5)

**Table 4:** Factors associated with infant mortality in Nepal in 2006 (unadjusted and adjusted odds ratio)

	NDHS 2006							
Variables	Unadjusted				Adjusted			
	COR	95%CI		*P* value	AOR	95%CI		*P* value
Community level factors								
Region				0.002				0.022
Far-Western	1.770	1.019	3.074	0.043	1.498	0.903	2.483	0.116
Mid-Western	1.931	1.233	3.024	0.004	1.818	1.061	3.118	0.030
Western	0.866	0.446	1.682	0.669	0.810	0.387	1.696	0.573
Central	0.985	0.631	1.539	0.948	1.053	0.632	1.754	0.842
Eastern	1.000				1.000			
Ecological Zone				0.004				0.004
Terai	0.553	0.300	1.020	0.058	0.687	0.384	1.229	0.204
Hill	0.361	0.188	0.692	0.002	0.425	0.216	0.834	0.013
Mountain	1.000				1.000			
Delivery Assisted				0.014				0.012
Some assistance	0.517	0.360	0.874	0.014	0.496	0.288	0.855	0.012
No assistance	1.000				1.000			
Socioeconomic Factors								
Maternal occupation				0.003				0.016
Unemployed	0.466	0.284	0.767	0.003	0.537	0.324	0.889	0.016
Employed	1.000				1.000			
Maternal Education				0.008				
No education	2.194	1.335	3.606	0.022				
Incomplete	1.677	0.878	3.205	0.116				
primary/Primary Incomplete Secondary/Secondary	1.000							
Paternal Education				0.025				
No education	2.199	1.240	3.898	0.007				
Incomplete	1.659	0.929	2.962	0.086				
primary/Primary	1.517	0.844	2.724	0.162				
Incomplete Secondary/Secondary Higher	1.000							
Maternal Literacy				0.026				
Unable to read at all	1.469	1.047	2.061	0.026				
Able to read parts or whole sentence	1.000							
Wealth Index				0.049				
Poorest	1.905	0.946	3.835	0.071				
Poorer	1.371	0.644	2.921	0.411				
Middle	1.845	0.847	4.019	0.122				
Richer	1.081	0.534	2.191	0.827				
Richest	1.000							
Biomass use				0.001				0.002
Relatively non-polluting	0.208	0.088	0.490	0.001	0.237	0.099	0.571	0.002
Relatively high polluting	1.000							
Proximate Factors								
Maternal factors								
Decision Making on own				0.012				
health care need								
Respondent alone	0.498	0.299	0.828	0.008				
Respondent and	0.796	0.514	1.234	0.306				
Husband/partner/other	1.143	0.767	1.701	0.509				
Husband/Partner Alone/Someone Else	1.000							
Decision Making capacity on money expenditure				0.019				
Respondent alone	0.203	0.071	0.581	0.003				
Respondent and	0.383	0.130	1.124	0.080				
husband/partner/other	0.370	0.140	0.980	0.046				
Husband/Partner Alone	1.000							
Someone Else								
Maternal BMI				0.067				
Underweight	2.693	1.161	6.246	0.021				
Normal range	2.459	1.131	5.345	0.023				
Overweight/Obese	1.000							
Mother’s age at marriage				0.093				
<16 years	2.208	1.016	4.797	0.045				
17–21 years	1.803	0.851	3.819	0.123				
>22 years	1.000							
Mother’s age at child birth				0.074				
<16 years	1.854	1.080	3.184	0.026				
17–21 years	1.449	0.941	2.231	0.092				
>22 years	1.000							
Infant factors								
Sex of child				0.419				
Male	0.875	0.633	1.211	0.419				
Female	1.000							
Birth order				0.003				
>4^th^ birth rank	0.754	0.507	1.121	0.162				
2^nd^ or 3^rd^ birth	0.535	0.375	0.764	0.001				
1^st^ birth rank	1.000							
Preceding Birth Interval				0.001				
<=24 months	2.240	1.592	3.153	0.001				
>24 months	1.000							
Succeeding Birth Interval				0.001				0.001
<=24 months	5.653	3.729	8.569	0.001	6.694	3.757	11.92	0.001
>24 months	1.000				1.000		5	
Baby’s size at birth				0.027				
Very large/larger than	0.516	0.304	0.877	0.015				
average	0.566	0.358	0.895	0.015				
Average	1.000							
Very small or smaller than average								
Delivery factors								
Place of delivery				0.027				
Home	1.781	1.108	2.863	0.017				
Health Facility	1.000							
Delivery assisted by				0.001				0.016
Professional	0.276	0.127	0.599	0.001	0.374	0.148	0.944	0.038
TBA	0.522	0.312	0.873	0.014	0.619	0.306	1.254	0.182
Combined	1.593	0.639	3.972	0.315	2.027	0.710	5.782	0.185
No assistance	1.000				1.000			
Mode of Delivery				0.060				
Normal delivery	3.520	0.945	13.108	0.060				
Caesarean Section	1.000							
Currently breastfeeding				0.001				0.001
No	1.773	1.333	2.358	0.001	2.650	1.928	3.645	0.001
Yes	1.000				1.000			

*Abbreviations*: *COR* crude odds ratio, *AOR* adjusted odds ratio

**Table 5:** Factors associated with infant mortality in Nepal in 2011 (unadjusted and adjusted odds ratio)

	NDHS 2011							
Variables	Unadjusted				Adjusted			
	COR	95% CI		*P* value	AOR	95% CI		*P* value
Community level factors								
Region				0.409				
Far-Western	1.498	0.995	2.254	0.053				
Mid-Western	1.135	0.739	1.744	0.561				
Western	1.271	0.826	1.956	0.274				
Central	1.176	0.716	1.933	0.520				
Eastern	1.000							
Ecological Zone				0.124				
Terai	0.653	0.424	1.004	0.052				
Hill	0.712	0.484	1.046	0.083				
Mountain	1.000							
Delivery Assisted				0.091				0.015
Some assistance	0.523	0.247	1.109	0.091	0.347	0.148	0.814	0.015
No assistance	1.000				1.000			
Socioeconomic Factors								
Maternal occupation				0.726				
Unemployed	1.073	0.721	1.598	0.726				
Employed	1.000							
Maternal Education				0.329				
No education	1.754	0.706	4.361	0.225				
Incomplete	1.521	0.587	3.940	0.387				
primary/Primary	1.273	0.488	3.320	0.621				
Incomplete Secondary/Secondary	1.000							
Paternal Education				0.004				0.010
No education	2.844	1.405	5.757	0.004	3.011	1.471	6.162	0.003
Incomplete	2.555	1.245	5.246	0.011	2.788	1.354	5.738	0.006
primary/Primary	1.676	0.815	3.446	0.159	1.756	0.909	3.389	0.093
Incomplete Secondary/Secondary Higher	1.000				1.000			
Maternal Literacy				0.017				
Unable to read at all	1.481	1.072	2.046	0.017				
Able to read parts or whole sentence	1.000							
Wealth Index				0.582				
Poorest	1.620	0.926	2.835	0.091				
Poorer	1.466	0.781	2.750	0.233				
Middle	1.527	0.824	2.830	0.178				
Richer	1.497	0.766	2.924	0.237				
Richest	1.000							
Biomass use				0.054				
Relatively non-polluting	0.594	0.349	1.008	0.054				
Relatively high polluting	1.000							
Proximate Factors								
Maternal factors								
Decision Making on own health care need				0.421				
Respondent alone	0.823	0.470	1.441	0.495				
Respondent and	0.657	0.389	1.110	0.116				
Husband/partner/other	0.817	0.495	1.349	0.428				
Husband/Partner Alone/ Someone Else	1.000							
Decision Making capacity on money expenditure				0.455				
Respondent alone	0.429	0.062	2.985	0.391				
Respondent and	0.498	0.069	3.601	0.488				
husband/partner/other	1.042	0.132	8.208	0.969				
Husband/Partner Alone Someone Else	1.000							
Maternal BMI				0.467				
Underweight	0.603	0.251	1.447	0.256				
Normal range	0.693	0.356	1.350	0.280				
Overweight/Obese	1.000							
Mother’s age at marriage				0.490				
<16 years	1.230	0.656	2.307	0.517				
17–21 years	1.001	0.543	1.845	0.999				
>22 years	1.000							
Mother’s age at child birth				0.127				
<16 years	1.623	0.933	2.824	0.086				
17–21 years	1.576	1.008	2.465	0.046				
>22 years	1.000							
Infant factors								
Sex of child				0.213				
Male	1.193	0.903	1.577	0.213				
Female	1.000							
Birth order				0.273				
>4^th^ birth rank	1.312	0.875	1.968	0.187				
2^nd^ or 3^rd^ birth	1.009	0.659	1.545	0.967				
1^st^ birth rank	1.000							
Preceding Birth Interval				0.001				0.038
<=24 months	2.121	1.459	3.084	0.001	1.941	1.036	3.635	0.038
>24 months	1.000				1.000			
Succeeding Birth Interval				0.001				0.003
<=24 months	4.162	2.579	6.717	0.001	3.215	1.505	6.866	0.003
>24 months					1.000			
Baby’s size at birth				0.003				0.025
Very large/larger than	0.399	0.233	0.684	0.001	0.170	0.047	0.624	0.008
average	0.569	0.365	0.886	0.013	0.717	0.333	1.546	0.394
Average Very small or smaller than average	1.000				1.000			
Delivery factors								
Place of delivery				0.316				
Home	1.230	0.820	1.846	0.316				
Health Facility	1.000							
Delivery assisted by				0.291				
Professional	0.482	0.224	1.038	0.062				
TBA	0.556	0.255	1.215	0.141				
Combined	0.448	0.126	1.597	0.215				
No assistance	1.000							
Mode of Delivery				0.029				0.022
Normal delivery	4.073	1.152	14.399	0.029	4.423	1.664	3.379	0.022
Caesarean Section	1.000				1.000			
Currently breastfeeding				0.002				0.001
No	1.618	1.190	2.202	0.002	2.382	1.674	3.390	0.001
Yes	1.000				1.000			

*Abbreviations*: *COR* crude odds ratio, *AOR* adjusted odds ratio

Tables 4 and 5 summarizes the identified factors associated with infant mortality for the NDHS 2006 and 2011, respectively. For NDHS 2006, among community level factors, the study found that infants born in the Mid-Western region had 82% higher odds of dying; the odds of death for infants born in Hilly region was reduced by 57% compared to those born in the Mountain region; and infants born to mothers who received some assistance during delivery had reduced the odds of death by 50% (Tables 4 and 5). Within only the group of socioeconomic level factors, the study revealed that infants born to unemployed mothers or born in families using relatively non-polluting cooking fuel had lowered odds of death (Tables 4 and 5). Considering proximate level factors specifically, infants who were born with a shorter than 24 months succeeding birth interval, and not breastfed were found to have significantly higher odds of death. On the other hands, the odds of mortality for infants who were delivered by the assistance of professionals reduced significantly.

For NDHS 2011, delivery assistance was the only community level factor that was significantly associated with infant mortality (Tables 4 and 5). Assistance during delivery (AOR = 0.35, 95% CI: 0.15–0.81, *p* = 0.015) was found to have protective effect for infants. For socioeconomic level factors, only paternal education was found to be significant negatively associated with infant mortality (*p* = 0.010). Regarding proximate level determinants, only some infant and delivery factors were found to be strongly associated with infant mortality (Tables 4 and 5). Infants who were born through normal delivery modes; who were born with a preceding or a succeeding interval of less than 24 months; who were born very small or smaller than average; or who were not breastfed were found to have significant higher odds of dying compared to their counterparts.

### Final factors associated with infant mortality (final overall hierarchical multiple logistic regression analysis Table 6)

**Table 6:** Overall significant adjusted odds ratio (AOR) for IMR in 2006 and 2011

Variables	2006				2011			
	AOR	95%CI		*P* value	AOR	95%CI		*P* value
Ecological Zone				0.004				
Terai	0.687	0.384	1.229	0.204				
Hill	0.425	0.216	0.834	0.013				
Mountain	1.000							
Preceding Birth Interval								0.022
<=24 months					1.941	1.036	3.635	0.022
>24 months					1.000			
Succeeding Birth				0.001				0.002
Interval	6.656	3.736	11.859	0.001	3.215	1.505	6.866	0.002
<=24 months >24 months	1.000				1.000			
Baby’s size at birth								0.015
Very large/larger than					0.170	0.047	0.624	0.008
average					0.717	0.333	1.546	0.394
Average Very small or smaller than average					1.000			
Delivery assisted by				0.016				
Professional	0.370	0.144	0.951	0.039				
TBA	0.589	0.277	1.254	0.168				
Combined	2.050	0.678	6.198	0.201				
No assistance	1.000							
Currently Breastfeeding				0.044				
No	1.618	1.014	2.580	0.044				
Yes	1.000							

AOR model for 2006 and 2011 was obtained after including all three final models (community, socioeconomic and proximate) through backward elimination

*Abbreviations*: *COR* crude odds ratio, *AOR* adjusted odds ratio

The final overall model identified ecological region, succeeding birth interval, currently breastfeeding and type of delivery assistance as the significant predictors affecting infant mortality for NDHS 2006 (Table 6). Infants who were born in Mountain region; who were born with a succeeding birth interval of less than 24 months (AOR = 6.66, 95% CI: 3.74–11.86, *p* = 0.001) and who were not breastfed (AOR = 1.62, 95% CI: 1.01–2.58, *p* = 0.044) had significantly higher odds of dying. However, the odds of mortality were reduced odds by 63% (AOR = 0.37, 95% CI: 0.14–0.95, *p* = 0.039) for those infants who were delivered through the assistance of professionals (doctors, nurses and midwives) (AOR = 0.37, 95% CI: 0.14–0.95, *p* = 0.039).

For NDHS 2011, three (all proximate level) infant factors were identified by the final model. Infants born with preceding birth intervals (AOR = 1.94, 95% CI: 1.04–3.64, *p* = 0.022) or succeeding birth intervals (AOR = 3.22, 95% CI: 1.51–6.87, *p* = 0.002) less than 24 months had significant higher odds of mortality compared to their counterparts. In addition, infants who were born very large or larger than average had significantly reduced odds (AOR = 0.17, 95%CI: 0.05–0.62, *p* = 0.008) of dying compared to those born very small or smaller than average.

## Discussion

This study explored and compared the associated risk factors of infant mortality using evidence from NDHS 2006 and 2011. The bivariate and multivariate regression models of this study found a number of significant predictors (Tables 4 and 5); however, they could not retain their significance in the final model (Table 6). Without losing any important association, the following discussion will be mainly based on the findings revealed by the final hierarchical overall model, which was built with a backward elimination regression approach with an inclusion of all possible significant predictors obtained from previous models.

### Findings from NDHS 2006 and 2011

Based on the final overall model (Table 6), infants born in hilly ecological region, delivery assistance by professionals and current breastfeeding status appeared as protective factors against infant mortality while succeeding birth interval of less than 24 months (2 years) was identified to be associated with the increased risk of infant mortality in the both study periods. Hence, both proceeding and succeeding birth intervals of less than 24 months (2 years) were associated with a significant increased risk of infant death, however, very large/larger than average birth size was negatively associated with infant mortality.

### Comparisons of the findings between 2006 and 2011 NDHS

Only succeeding birth interval was the common factor for both surveys (Table 6). Ecological region, type of delivery assistance and current breastfeeding status were found to have a significant impact on infant mortality only in 2006 survey however they didn’t show their significant impacts in 2011 survey. Preceding birth interval and baby birth size emerged as new significant factors in 2011 survey. Interestingly, different levels factors (community level, proximate level factors (delivery and infant) affected infant mortality in 2006 while in 2011 only proximate level factors, more specifically, infant factors played an important role on infant mortality (See Table 6). No socio-economic level or maternal factors were found to be significant for both surveys based on the final overall model.

### Discussion of the findings between 2006 and 2011 NDHS

#### Ecological region

In our study, ecological region was found as a significant predictor for 2006 survey only, its less important impact for 2011 survey could be attributed to improved transportation, availability of health care facilities and increased human resources in health, although not reach to the expected level, in Nepal [23]. Dev [4] reported that infants born in Mountain region in Nepal had 42% increased odds of mortality within the infancy period compared to those in the Terai region. Infants born in Hill and Terai had significantly achieved 55% reduction in mortality between 1996 and 2011 compared to those born in the Mountain region [5]. Based on the findings of Baral, Lyones, Skinner and van Teijlingen [24] mothers from the central and Terai region were more likely to utilise health care services compared to those from Far Western region and Mountain areas. The study further reveals that the majority of people living in mountainous zone (far and mid-western region) had lower access to healthcare services and had relatively poor standard of living. Furthermore, access to health care facilities is limited due to poor transportation and difficult geographical terrain [10]. Human development index (HDI) of the people living in mountainous Mid-Western and Far-Western regions was 0.398, extensively lower compared to those living in Kathmandu valley, which was 0.622 in 2011 [25]. Another study reported an insignificant association between regional variation and infant mortality [26]. Some other studies have identified that eco-developmental region was significantly associated with infant mortality [4, 7, 18].

#### Delivery factors

Regarding health service coverage, delivery assistance showed its important effect when only community level factors were adjusted and it lost its significance in the final overall model (Tables 4 and 5). A similar study in Indonesia also found delivery assistance as a protective factor for infant mortality [26]. Unassisted births had a greater risk of infant mortality compared to those who had some assistance [24]. In Nepal, access to delivery services especially comprehensive obstetric care is inadequate because of limited human resources and extreme geographical locations [24]. As a consequence, the majority of infants who are born with birth complications like birth asphyxia and prematurity do not get timely treatment which leads to their death [27]. Limited access to caesarean section facility could be a possible explanation for higher infant mortality among those normally delivered [13].

The final overall model confirmed that type of delivery assistance was a significant predictor (Table 6) for 2006 survey and it failed to find the similar significant association for NDHS 2011. This could be attributed to more implementations of maternal and child health related policy and strategies during 2005 to 2009 [27]. Those infants who were delivered with professional assistance (doctors, nurses and midwives) were less likely to die. Professional assistance during delivery was found to be a protective predictor against the infant mortality. A similar study conducted in Indonesia also reported consistent findings with this study [28]. In addition, mothers living in urban areas were advantaged with delivery assistance reporting that 51% of urban births were assisted by professionals while only 14% of births were assisted in rural areas [24]. Those infants born to mothers who were able to make decisions on her own had reduced odds of infant mortality compared to those whose decisions were made by someone else. Some studies reported significant association between infant mortality and mother’s decision-making capacity [29, 30]. Nonetheless, a cross-sectional study conducted in India could not find any significant association, although mothers’ decision-making capacity was found to affect the quality of child care and access to health information [31]. Neupane and Doku [32] and several other studies also had consistent findings with our result [20, 28]. Nepal government endorsed a skilled birth attendant policy in 2006 and maternal incentive schemes in 2005 to encourage women to utilise maternity care services [13, 27]. The policies implemented during that period increased service coverage in 2011 compared to 2006 to some extent [13]. Our study further identified that IMR was higher among those who did not utilise the available facilities. Research has reported that geographical difficulties and poor transportation are possible barriers for the underutilisation of maternal health services in rural areas [20, 24, 28].

Although it lost its significance in the final overall model, normal delivery was identified to be associated with an increased likelihood of infant death for 2011 survey. Contrastingly, Titaley, Dibley and Roberts found a lower risk of mortality among infants who were delivered normally compared to those delivered through caesarean section [26]. However, the finding was not significant in their study. In addition, another study conducted by the same authors reported different findings that an increased risk of dying among neonates born through normal delivery [26]. In Nepal, access to delivery services especially comprehensive obstetric care is inadequate because of limited human resources and extreme geographical locations [24]. As consequence, the majority of infants who are born with birth complications like birth asphyxia and prematurity do not get timely treatment which leads to their death [27]. Limited access to caesarean section facility could be a possible explanation for higher infant mortality among those normally delivered [13]. Similar to ours, other studies also could not find any significant association between maternal age and infant mortality [26, 32].

#### Birth intervals

Interestingly, none of the maternal factors were found to be significant predictors of infant mortality however, infant factors played very important role on infant survival, particularly for 2011 survey. Birth intervals (preceding and succeeding)^1^ were positively correlated with infant survival (Table 6). Those infants born to a short (less than 24 months) preceding or succeeding birth interval were at higher risk of mortality compared to those born with a longer birth interval (more than 24 months). Several epidemiological studies conducted in developing countries supported the findings for birth interval and infant mortality [20, 26, 33]. Spacing between pregnancies is an influential factor of infant mortality. A study illustrated that women with a short birth interval between pregnancies do not get sufficient time to maintain their normal body structure and nutritional status [26]. The birth spacing between pregnancies strongly correlates with child survival in Nepalese population and other developing countries [12]. According to the Indonesian study, a short birth interval increased the odds of infant death during the neonatal period [34]. Those infants born with less than 2 years (<24 months) preceding or succeeding birth interval had a greater risk of dying compared to those born with more than 2 years’ interval.

### Baby birth size

Another known significant predictor of infant mortality was baby’s size at birth in literature and this study confirmed that those infants who were born with very large or larger than average size at birth had significantly lower odds of dying compared to those born with very small or smaller than average birth size. A study conducted in India among neonates revealed that mortality was highest among newborns whose birth size was smaller than average [35]. Another epidemiological study also found a similar association reporting lower odds of dying among infants born with average or larger than average birth sizes [36].

### Breastfeeding

This study also confirmed that current breastfeeding (during the time of survey) was a protective predictor for infant survival. Those infants who were not currently being breastfed had 2.65 times higher risk of dying compared to their counterparts. Studies have identified that breastfed children were more likely to be protected from several infections and mortality [37]. A meta-analysis conducted between 1966 and 2009 found breastfeeding to be protective against Sudden Infant Death Syndrome (SIDS). The same study further reported that infants who were breastfed for any duration were more likely to be protected against mortality [38]. Khanal, Sauer and Zhao [37] identified breastfeeding as one of the protective factor for infant survival. Other researchers further identified consistent findings that breastfeeding reduces the risk of major infections as well as SIDS during infancy [38, 39].

#### Other important factors

Several policies like Maternal Incentive Scheme and Free Delivery Services, addressing transportation and health service coverage issues related to maternal and child health were implemented during that period (2006 and 2011), which could be a potential explanation for the insignificant impact of eco-developmental regions on infant mortality [27]. Our study identified significantly higher odds of infant mortality among those infants whose mothers did not receive antenatal services, however, the association lost significance after controlling for all other community level factors (Tables 4 and 5).

Based on the final overall model, paternal education and maternal literacy status were not found to be significantly associated with infant mortality in both surveys. In Nepalese patriarchal society, the father contributes the major input to family’s economic status and dominates decision making. Hence, the significant association between infant mortality and father’s education indicated a protective effect on infant’s survival [40]. Similar studies conducted in Indonesia and in Nepal reported insignificant association between maternal educational status and infant mortality [32, 34], which is consistent with our findings. A study conducted in Nepal found wealth index as one of the significant socioeconomic predictors of infant mortality [20]. However, our study found an insignificant association between the wealth index and infant mortality. A consistent finding was also reported by one of the survival analysis conducted in Nepal [34]. Although odds of dying for those infants born to families using relatively non-polluting cooking fuel was lower compared to their counterparts, biomass use was found not to be associated with infant mortality for NDHS 2011 in our study [41, 42].

To sum up, this study used the data from two DHS period (2006 and 2011) to identify and compare the significant predictors of infant mortality in the Nepalese context. Succeeding birth internal was the only factor which was common in both these periods. Other factors significant in either of these period were hilly ecological region, delivery assistance by professionals, current breastfeeding status and birth size. Most of the study findings are consistent with the existing literature from around the world in infant mortality. This study thus helps to establish these factors in the Nepalese context. Additionally, this study suggest variation in the factors significantly affecting infant mortality between the two DHS periods such as birth size which was significant in 2011 but not in 2006. Study incorporating the data from the upcoming NDHS 20016 might provide more clarity regarding this.

## Conclusion and recommendations

This study found four factors in NDHS 2006 and three factors in NDHS 2011 that were significantly associated with infant mortality based on the final overall model. For NDHS 2006, infants who were born in hilly region; who were born with a succeeding birth interval of ≥ 24 months; who were delivered with professional assistance; and who were being breastfed, had lower odds of dying. For NDHS 2011, infants who were born with a preceding or succeeding birth interval of >24 months; and who were born with a larger or larger than average size had significant lower odds of dying. Succeeding birth interval was the only common factor significantly associated with infant mortality for both the study periods.

Infant mortality is still significantly high in Nepal based on the two nationally representative survey data, NDHS 2006 and NDHS 2011, implying that there is an urgent need for the country to implement more targeted public health interventions which can accelerate the decrease in infant mortality with an aim to improve the infant survival rate. The study revealed that geographical difficulties and service coverage had influenced infant mortality; therefore, it is essential to increase the access and availability of health care services in hard-to-reach areas. Inter-sectoral collaboration between health and other sectors in areas such as parental literacy and indoor air pollution can bring better result. We recommend that efforts on increasing the number of health facilities along with skilled health care providers, and providing accessible basic and comprehensive emergency obstetric care should be encouraged. Furthermore, this study found that birth interval was the strongest predictor in both surveys. Therefore, intervention programs should particularly focus on addressing birth spacing using efficient strategies like strengthening of family planning programs in the community. Similarly, study incorporating the data from the upcoming NDHS 2016 is recommended to establish the significant predictors in the Nepalese context.

### Strengthens and limitations of the study

One of the strengths of this study is the use of well-documented data from the NDHS 2006 and 2011, which are nationally representative surveys with a high response rate (99%). Questionnaires were internationally validated and used standardised methods of data collection with larger sample size. Both surveys had a large sample size which allowed for the inclusion of a wide range of variables that are associated with infant mortality and permitted insight examination of multiple predictors, possible interactive association and confounding effects. Furthermore, this study categorised a wide array of potential factors into three different groups under conceptual framework including: community, socioeconomic and proximate level factors, and helped to further identify the most significant factors within and between different levels. This study used complex sample analysis, which accounts for the sampling weight due to multistage stratified sampling used in both surveys, to obtain accurate estimation for standard errors and confidence intervals. This study compared and examined the difference in factors associated with infant mortality in Nepal between the 2006 and 2011 national surveys, which has not been reported yet. Hence, the comparison provides evidence-based recommendations for further studies, interventions planning and policy decisions making.

As DHS are derived from cross-sectional surveys, such data might be subjected to recall bias. The association of infant mortality with factors drawn from statistical analysis might lack a temporal relationship because of the nature of the study design. Furthermore, this study only included singleton live-births 5 years preceding the surveys. Another limitation of this study is regarding small number of observations in some categories defined by several independent variables, where recoding/regrouping was not possible. These sparse observations caused some computational difficulties in regression analysis and some important variables such as maternal antenatal visit, birth order, maternal age and BMI etc. might have been missed as significant predictors in this study.

## Abbreviations

AIDS: Acquired immune deficiency syndrome
AOR: Adjusted odd ratio
BMI: Body mass index
CI: Confidence interval
COR: Crude odd ratio
DHS: Demographic and health survey
HDI: Human development index
HIV: Human immunodeficiency virus
IMR: Infant mortality rate
MDG: Millennium development goal
NDHS: Nepal demographic and health survey
PQLI: Physical quality of life index
SIDS: Sudden infant death syndrome

## Footnotes

1 Preceding: Before Succeeding: Subsequent.
